# Effectiveness of Self-Management Programs Among Athletes With Patellofemoral Pain Syndrome: Protocol for a Systematic Review

**DOI:** 10.2196/58340

**Published:** 2024-11-01

**Authors:** Ameen Masoudi, Nomzamo Chemane, Ushotanefe Useh, Bashir Bello, Nontembiso Magida

**Affiliations:** 1 Physiotherapy Department University of KwaZulu-Natal Durban South Africa; 2 Lifestyle Diseases Research Entity Faculty of Health Sciences North West University Mafikeng South Africa; 3 Physiotherapy Department Faculty of Allied Health Sciences Bayero University Kano Nigeria; 4 Department of Physiotherapy Faculty of Healthcare Sciences University of Pretoria Pretoria South Africa

**Keywords:** patellofemoral pain syndrome, athletes, self-management, chronic pain, pain management, systematic review

## Abstract

**Background:**

Patellofemoral pain syndrome is a highly prevalent overuse knee injury in athletic populations associated with pain and functional limitations, exacerbated by activities such as running, pivoting, cycling, and jumping. Self-management programs empowering athletes to take an active role in controlling their symptoms for chronic musculoskeletal conditions such as patellofemoral pain syndrome have grown in popularity. However, the efficacy of self-management programs specifically for athletes with patellofemoral pain syndrome is unclear due to limited and heterogeneous evidence.

**Objective:**

The systematic review study will evaluate the effectiveness of self-management programs on pain and function, in athletes with patellofemoral pain syndrome.

**Methods:**

PubMed/MEDLINE, Cochrane Library, CINAHL (Cumulative Index to Nursing and Allied Health Literature), and PsycINFO databases will be systematically searched using terms related to “patellofemoral pain syndrome,” “self-management,” and “athletes.” Interventional studies that are randomized and nonrandomized controlled trials will be included, comparing self-management programs to other treatments or control conditions among athletes with patellofemoral pain syndrome. Four reviewers will independently screen studies, extract data using the COVIDENCE software, and assess the quality of the study and evidence using the Pedro scale of risk of bias tool and GRADE approach, respectively. If feasible, a meta-analysis will be performed using the RevMan (version 5.4; the Cochrane Collaboration).

**Results:**

The systematic review is currently in the search phase, with the authors refining search strings for the selected databases. The final search strings are expected to be ready by March 2024, and the review is projected to be completed by July 2024.

**Conclusions:**

This systematic review protocol outlines a rigorous methodology for evaluating the effectiveness of self-management programs among athletes with patellofemoral pain syndrome. The findings will inform clinical practice and guide the development of tailored interventions to optimize outcomes for athletes with patellofemoral pain syndrome.

**Trial Registration:**

PROSPERO CRD42023492746; https://tinyurl.com/c5jze9ca

**International Registered Report Identifier (IRRID):**

PRR1-10.2196/58340

## Introduction

### Background

Patellofemoral pain syndrome (PFPS) is a common and prevalent musculoskeletal disorder affecting athletes, particularly those engaged in repetitive knee flexion, such as running, jumping, and cycling. Sports participation often entails rigorous training regimens and competitive demands, leading to overuse injuries and biomechanical stress on the patellofemoral joint. Additionally, athletes may exhibit specific risk factors predisposing them to PFPS, such as quadriceps muscle weakness, hip muscle dysfunction, patellar malalignment, and foot biomechanical abnormalities [1]. These factors can disrupt patellofemoral joint mechanics, alter load distribution, and contribute to the onset and persistence of PFPS symptoms among athletes.

The prevalence of PFPS is about 25% of knee injuries seen in sports rehabilitation clinics, with incidence rates ranging from 9% to 40% among active individuals in United States, England, and Belgium [2]. Despite its high prevalence, effective management strategies for PFPS remain elusive, often leading to prolonged pain, functional limitations, and decreased athletic performance [3].

The management of PFPS typically involves a multimodal approach, including rest, activity modification, physical therapy, and, in some cases, surgical intervention [4]. However, recent emphasis has been placed on self-management strategies to empower athletes to take an active role in their recovery process [5]. Esculier et al [6] affirmed that self-management programs (SMPs) aim to educate individuals about their condition, promote self-efficacy, and provide tools and techniques for symptom management and functional improvement. While there is growing interest in self-management approaches for PFPS, the evidence supporting their effectiveness among athletes remains limited and heterogeneous [7].

Several studies have investigated the efficacy of various self-management interventions, including exercise therapy, taping, bracing, and education, but findings have been inconsistent, and the optimal combination and delivery of these interventions remain unclear [8-10]. This systematic review will, therefore, aim to synthesize the current evidence regarding the effectiveness of self-management programs among athletes with PFPS. By critically evaluating the existing literature, we seek to identify the most effective self-management strategies for pain reduction, functional improvement, and return to sport among this population. Cowan et al [1] stated that such systematic review findings provide insights that are essential for informing clinical practice and optimizing outcomes for athletes with PFPS. This is important as less is known about the self-management program and its impact on individuals with chronic dysfunction. Similarly, there is limited knowledge of how other stakeholders, such as caregivers, health experts, and researchers, view self-management programs in the context of disability, chronic health conditions, and assistive technologies [11]. Hence, the present study is proposed to evaluate the existing evidence on the effectiveness of SMP in reducing knee pain and improving function among athletes with PFPS.

### Objectives

This study will identify, appraise, and synthesize the existing evidence on the effectiveness of self-management programs among athletes with patellofemoral pain syndrome.

## Methods

### Overview

The PRISMA (Preferred Reporting Items for Systematic Reviews and Meta-Analyses) protocol will be used to design the systematic review as described by Souto et al [12].

### Eligibility Criteria

#### Inclusion Criteria

##### Study Design

Randomized clinical trials (RCTs) and non-RCTs that compare self-management program (SMP) as an intervention with a control group (placebo, adjunctive treatment, or alternative therapy alone) will be eligible for the study.

##### Type of Population

The current systematic review will include articles involving athlete participants clinically diagnosed with PFPS, using the main criteria of pain around or behind the patella, which increases by running, jumping, squatting, kicking, jogging, riding, and other sporting activities.

##### Type of Intervention

Self-management programs concerning PFPS treatment interventions, including nonpharmacological interventions such as patellofemoral knee orthoses (bracing), patellar taping, foot orthoses, weight loss, psychological therapy, exercises, education, behavioral changes, and other complementary therapies will be included in the study.

##### Comparison of Interest

Studies with or without a comparator group will be considered. Comparators may include PFPS standard care, no intervention, placebo, or an alternative treatment.

##### Outcome Measures

Studies that assess pain intensity and functional ability as primary outcomes through validated measures within the PFPS population will be included. The secondary outcome will be compliance and adherence to self-management programs, treatment satisfaction, no recurrence of PFPS, and no adverse effects. Both short-term and long-term assessments would be considered.

### Exclusion Criteria

Review studies and protocols will be excluded in this study. Studies that examine other knee conditions such as osteoarthritis, patella dislocation, and sports injuries (anterior cruciate ligament, meniscus, and unhappy triad), leading to ligamentous instability or knee pain will be removed. Studies of participants with previous knee, hip, ankle, or low back surgeries will be excluded.

### Search Strategy

#### Overview

The search strategy and eligibility criteria will be based on the PICOS (Participant, Intervention, Design, Comparator, Outcomes and Study design) framework. The search will include a combination of keywords and MeSH (Medical Subject Headings) terms related to PFPS, self-management programs, and pain measurements. Keywords used in the search strategy adhere to each of the key characteristics of the PICO framework. These include patellofemoral pain as the participant (“Patellofemoral pain syndrome,” Anterior Knee Pain Syndrome) self-management as the intervention (“Self-management,” “Exercise therapy” OR “Biofeedback”), experimental controlled studies as the design and research type (“RCT”), and pain and function as the outcomes (“pain intensity” and “Pain Severity”). The Boolean operators “AND” and “OR” will be used to refine the search strategy. A sample search strategy for PubMed (MEDLINE) is outlined below:

1. (“Anterior Knee Pain Syndrome” OR “Patellofemoral Syndrome” OR “Patellofemoral Pain” OR “Patellofemoral Pain Syndrome”) AND
2. (“Self-management program” OR “Exercise therapy” OR “Biofeedback” OR “Muscle strengthening” OR “Education”) AND
3. (“Pain Intensity” OR “Pain Severity” OR “Pain Measurement” OR “Pain Assessment”)

The search strategy will involve an initial search of electronic databases, followed by the analysis of keywords in the titles and abstracts of each database search. The search will then continue to the reference lists of selected articles to identify additional studies not located through the electronic database search. The strategy will aim to locate published articles—gray literature and unpublished articles will be excluded from the review. The search will not be limited to specific period but it will include all articles published from inception of the database till the start of the search in March 2024. The search strategy will be adapted for each electronic database. The search strategy for each data source will be developed by 4 researchers (BB, NC, NM, and AM). Language will not be used to filter publication during the search.

#### Information Sources

A literature search for experimental controlled studies will be conducted on the following electronic databases: PubMed (MEDLINE), Cochrane Library, CINAHL (Cumulative Index to Nursing and Allied Health Literature), and PsycINFO. These databases have been chosen as they encompass nursing, medicine, social sciences, and psychology literature, which are deemed the most appropriate for answering the research question concerning self-management of patellofemoral pain.

### Data Management

The search results will be loaded into COVIDENCE, a systematic review reference management program, to find and eliminate duplicates.

### Study Selection

The selection process will be performed by the 4 independent reviewers (BB, NC, NM, and AM) who will screen the titles and abstracts. The researchers will independently apply the inclusion and exclusion criteria after thoroughly reading the selected studies. In disagreements, consensus will be sought; however, a fifth reviewer (UU) will be consulted if disagreement persists. The reviewer (AM) will contact the study authors if the complete information is unavailable. The study will be excluded if the authors cannot provide the full article or fail to reply to the request after 3 attempts.

### Data Extraction

After selection of the primary studies, the 4 reviewers (BB, NC, NM, and AM) will work independently. Consensus will resolve disagreements; should they persist, an independent reviewer (UU) will be consulted. Data relevant to the research question and predefined criteria will be extracted from the selected studies. Extracted data will include author, population, age range, study design, participant characteristics, details of the self-management intervention, controls, outcomes measured, adverse effects, results, and conclusion.

A pilot testing of the data extraction form will be conducted using a subset of studies for accuracy purpose. Reviewers will meet regularly to discuss and resolve any uncertainties or discrepancies in the data extraction process. The extracted data will be cross-checked between reviewers to minimize errors and enhance reliability. The extracted data will be saved and stored in COVIDENCE allowing easy access and retrieval during the synthesis and analysis stages. A version control system will be implemented to track any updates or modifications made during the data extraction process. Authors will be contacted for missing or unclear information. If data cannot be obtained, the potential impact of missing data will be addressed and reported in the systematic review. The collected data will be reported transparently and comprehensively in the final systematic review, including tables summarizing critical characteristics of the included studies and their findings. These processes are aimed to enhance the reliability and validity of the systematic review’s findings.

We aim to start reviewing articles in March 2024 and completing data extraction in June 2024.

### Data Analysis

The results will be synthesized through a narrative approach using qualitative thematic analysis of the studies via line-by-line coding of the results and discussion sections to identify contextual information on self-management of patellofemoral pain, clustering the coded data to generate “descriptive themes,” identifying “analytical themes” about the data captured in the descriptive themes [13]. These themes will be relevant to the key aim of this study.

When appropriate, all self-management program effects will be converted to standardized mean difference for ease of comparison. For each program, pooled standardized mean difference values and measures of heterogeneity (such as I2 and assessment of publication bias) will be calculated using the random-effects meta-analysis model. The heterogeneity may come from varied designs, participant demographics, interventions, and result measures. If substantial heterogeneity exists, sensitivity analyses will be conducted to explore potential sources. The “R” program will be used for data synthesis. A significance level of α=.05 will be applied to determine statistical significance. The general quality of evidence will be evaluated using the GRADE system to provide a transparent evaluation of the strength of the findings. The selection process, including the number of studies screened, reasons for exclusion at each stage, and the included studies, will be reported as PRISMA flow diagram.

### Quality Appraisal

The assessment of bias for the studies included will be conducted using the PEDro scale and Cochrane risk of bias tool (if needed). The reliability of this tool is fair to good [14]. While the scale comprises 11 items, the inclusion of eligibility criteria specifications will be omitted from the final score, resulting in a scale range of 0-10. Each affirmative answer will receive one point, which will be added to obtain the final score [14]. The evaluation of studies listed in the PEDro database will be upheld, while nonindexed studies will undergo independent assessment by 2 reviewers (BB and AM). Ratings will categorize studies as having low risk if they score ≥7/10, moderate risk (4-6/10), or high risk of bias (≤3/10) according to this scale [15].

### Missing Data

If feasible, we will reach out to authors to acquire any absent data, particularly information crucial for conducting a meta-analysis. If authors are unable to furnish the missing data or do not respond after 3 attempts, the study will be excluded from subsequent statistical analyses.

Regarding documentation of significant protocol alterations, this section will log the date of each amendment along with a description of the modification and its rationale. These changes will be recorded without integration into the protocol itself.

## Results

This systematic review protocol was developed in November 2023 to assess the effectiveness of SMP for athletes with PFPS. To our knowledge, this will be the first systematic review focused specifically on SMPs for PFPS in athletes. The review aims to synthesize evidence on the effects of SMPs on outcomes such as pain and function, in athletes with PFPS. Upon completion (anticipated by June 2024), the outcome of this study will be submitted for publication in an academic journal that uses expert peer review process to evaluate research manuscripts.

Findings of the current study will fill the knowledge gap in front of researchers, physiotherapists, and athletes with patellofemoral pain syndrome as well regarding the effective and evidence-based self-management strategies that can be used by the patients or advised by the practitioners to their patients to reduce their pain and disability.

## Discussion

### Overview

It was hypothesized that there will be enough literature for both qualitative and quantitative synthesis and analysis that shows positive effects for the self-management programs on treatment of pain and disability in athletes with patellofemoral pain syndrome. The aim of this systematic review protocol will be to evaluate the current evidence on SMPs for treating PFPS in athletes. This review will synthesize evidence on the effects of SMPs on outcomes such as pain and function in athletes with PFPS.

### Principal Findings

Previous RCTs and systematic reviews have demonstrated the effectiveness of some self-management strategies in improving clinical outcomes in general population with patellofemoral pain [3,6,8-10]. Multiple studies have examined the effectiveness of SMPs for reducing pain and improving function in athletes with PFPS [16,17]. However, there is a lack of research collectively evaluating experimental controlled studies that explore how athletes with patellofemoral pain can self-manage themselves and the impact of this self-management on their pain and disability.

### Strengths and Limitations

The comprehensive approach of this systematic review gives it its strength. PICO framework will guide the eligibility criteria and search strategy. Search terms and strategies will be determined with the help of librarian and from PubMed MeSH terms. The review will follow the PRISMA checklist to report and synthesize the systematic evidence. The quality of included studies will be assessed using the 2 most recommended and strong checklist tools: PEDro and Cochrane. Data extraction will be performed by 5 independent researchers, and another reviewer will resolve any disagreement if present. Furthermore. this review will not limit search by year of publication or language. However, this review is limited by excluding grey literature, which may affect results and conclusions.

The discussion will summarize the components of SMPs that were effective for athletes with PFPS based on the studies reviewed. This systematic review will summarize the components of SMPs and clarify the evidence of their effectiveness in reducing pain and disability in athletes with PFPS. Based on the findings, this will inform future research, such as improving the quality of future studies, increasing treatment duration, improving adherence, and so on. It can also inform clinical practice recommendations.

## Abbreviations

CINAHL: Cumulative Index to Nursing and Allied Health Literature
MeSH: Medical Subject Headings
PFPS: Patellofemoral pain syndrome
PICOS: Participant, Intervention, Design, Comparator, Outcomes and Study design
PRISMA: The Preferred Reporting Items for Systematic Reviews and Meta-Analyses
RCT: randomized controlled trail
SMP: self-management program

## Data Availability

All data will be available via contacting the corresponding author upon request.
